# Role of Epstein-Barr Virus and Human Papillomavirus Coinfection in Cervical Intraepithelial Neoplasia in Chinese Women Living With HIV

**DOI:** 10.3389/fcimb.2021.703259

**Published:** 2021-09-07

**Authors:** Min Feng, Rufei Duan, Yang Gao, Han Zhang, Youlin Qiao, Qihan Li, Fanghui Zhao

**Affiliations:** ^1^Institute of Medical Biology, Chinese Academy of Medicine Sciences and Peking Union Medical College, Kunming, China; ^2^Department of Gynecology, The Third Affiliated Hospital of Kunming Medical University/Yunnan Cancer Hospital/Yunnan Cancer Center, Kunming, China; ^3^Department of Cancer Epidemiology, National Cancer Center/National Clinical Research Center for Cancer/Cancer Hospital, Chinese Academy of Medical Sciences and Peking Union Medical College, Beijing, China

**Keywords:** Epstein-Barr virus, human paillomavirus, coinfection, host gene expression, cervical intraepithelial neoplasia (CIN)

## Abstract

Given that only a small percentage of human papillomavirus (HPV)-positive women develop cancer, HPV is necessary but insufficient for carcinogenesis. Mucosally transmitted viral cofactors appear to contribute to HPV-related cervical cancer, such as Epstein-Barr virus (EBV), but previous studies have shown inconsistent outcomes. The exact role of EBV in cervical cancer remains unclear, and more studies are needed to determine its involvement. In this study, we describe the prevalence of EBV and HPV coinfection in HIV-positive women and explore how abnormal host immune status induced by viral coinfections modulates epithelial gene expression. We found a significant correlation between EBV-HPV coinfection and the incidence of high-grade cervical intraepithelial neoplasia (CIN2+). RNA sequencing indicated that CIN tissues coinfected with EBV and HPV led to significant changes in the gene expression of epithelial differentiation and development compared to normal tissues with HPV infection alone. In particular, several differentially expressed genes (DEGs) are closely associated with cancer, such as CACNG4, which was confirmed to be upregulated at both the mRNA and protein levels. Therefore, these findings provide some evidence that EBV may act as a cofactor or mediator in HPV-related cervical cancer. Specific genes or proteins, such as CACNG4, may serve as biomarkers that can risk stratify patients based on pathological changes in the cervix.

## Introduction

Sexually transmitted infections (STIs) can cause cancer or have serious reproductive health consequences (WHO, 2018), especially in women. Human papillomavirus (HPV) infection is the most prevalent STI in the female population (Satterwhite et al., 2013), with an estimated prevalence of 11-12% worldwide (Forman et al., 2012). Cervical cancer is the main HPV-related cancer, and according to the WHO, there were an estimated 570,000 new cases worldwide in 2018 (WHO). Although the etiological role of HPV in cervical cancer is well established, more than 90% of HPV infections can be cleared within a few months to 2 years without any intervention (Moscicki et al., 2006). Only 10% of women with high-risk HPV will develop long-lasting infections that put them at risk for cancer (Centers for Disease Control and Prevention, 2020), indicating that HPV is necessary but may not be sufficient for carcinogenesis. In addition to HPV infection, several biological and environmental cofactors have been implicated in the development of cervical cancer, such as immune status and coinfection with other sexually transmissible pathogens (Castellsague et al., 2002; Bosch and de Sanjose, 2007). Detection of viral cofactors involved in malignancy progression can be used for HPV-related cervical cancer intervention.

Similar to HPV, Epstein-Barr virus (EBV) is a mucosally transmitted pathogen that is widespread in the human population (Higgins et al., 2007). This virus is able to persist in the human host through the establishment of a lifelong latent infection, which could contribute to host immune modulation (Djaoud et al., 2017). EBV can infect cervical epithelial cells (Sixbey et al., 1983), and viral DNA, RNA or proteins of EBV have been detected in exfoliated cells or tissues from cervical cancer or intraepithelial neoplasia (CIN) lesions (Khenchouche et al., 2013; Marinho-Dias et al., 2013). A correlation between EBV infection and abnormal cervical cytology has been shown; on one hand, the prevalence of EBV positivity increased with lesion severity (Silver et al., 2011; Aromseree et al., 2015); on the other hand, CIN or cervical carcinoma occurs more often among EBV positive women than women without EBV infection (Cameron et al., 2018; de Lima et al., 2018). Recently, one study (Cameron et al., 2020) from a cohort of HIV-seropositive women, reported an approximately fourfold increased risk of abnormal cervical cytology in women with cervical EBV and high-risk HPV compared to women with high-risk HPV alone, suggesting that cervical EBV appears to predict a greater risk of cervical dysplasia in HIV-infected women with a high-risk HPV infection. Although a few studies (Kahla et al., 2012; Blanco et al., 2020) have provided explanations for how EBV can act as a risk factor for HPV persistence and tumor development, the exact role of EBV in cervical cancer remains unclear. In addition, the opposite result has also been reported, showing a lack of association between EBV infection and HPV-related cervical cancer (Ammatuna et al., 2000). Therefore, more studies are needed to determine whether EBV has a role in cervical cancer or acts only as an opportunistic pathogen.

In contrast to people with a normal immune system, women living with HIV are at high risk of acquiring oncogenic HPV and are also susceptible to persistent and reactivated HPV infection and to developing cancer (Ahdieh et al., 2001; Camargo et al., 2014; Jin et al., 2017). In addition, EBV incidence is also higher in the HIV-infected population (Miller et al., 2006; Jin et al., 2017). Therefore, the increased comorbidity among these sexually transmitted viruses in HIV-positive individuals may provide a better model to explore the possible mechanism of coinfection-associated malignancies. In this context, we detected viral DNA in exfoliated cells from cervix to describe the prevalence of coinfection between EBV and HPV in HIV-positive women and to determine the association between viral coinfection and CIN using mRNA sequencing (mRNA-seq). If EBV is a risk factor for HPV-related cervical cancer, we would expect that certain alterations in genes or proteins under specific coinfection conditions can be found to be potential predictive biomarkers for tumorigenesis in cervical disease.

## Materials and Methods

### Study Population and Procedure

**Figure 1:**
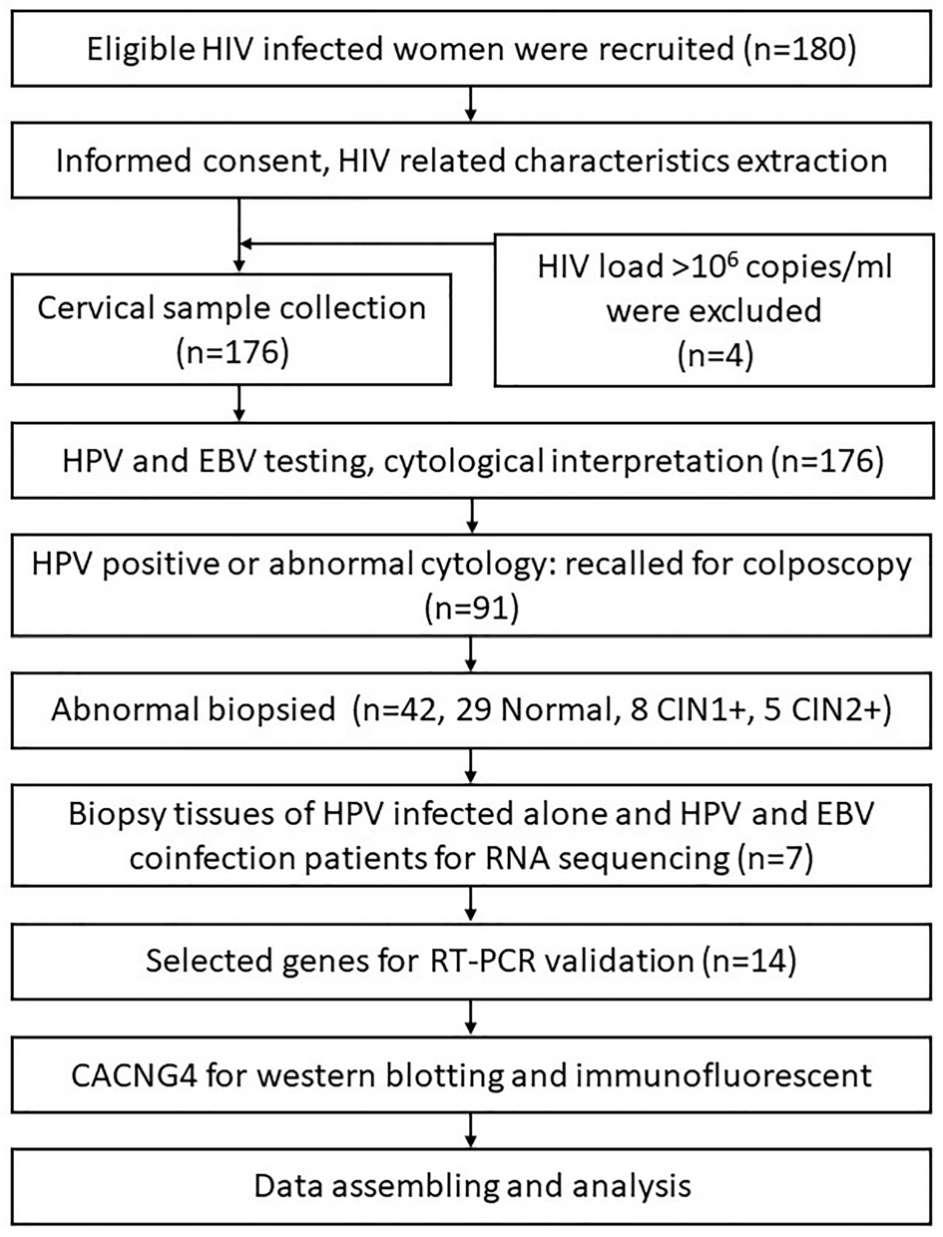
A summary of the study procedure.

Participants were recruited from the AIDS Antiretroviral Therapy Department of the Third People’s Hospital of Kunming, Yunnan Province, China, between February and November 2019. The inclusion criteria were as follows: 1) HIV-positive women with ongoing antiretroviral therapy, 2) age of 18-64 years, 3) sexually active, 4) ability to both physically and mentally undergo cervical sampling and colposcopy examinations, and 5) intact cervix and no current pregnancy. The study protocol was approved by the Ethical Review Committees of National Cancer Center/Cancer Hospital Chinese Academy of Medical Sciences and Peking Union Medical College, Beijing, China. All participants provided informed consent. A summary of the study procedure is shown in **Figure 1**.

### Sample Collection, HPV DNA Testing, and Cytology

Trained gynecologists inserted cytology brushes and collected exfoliated cervical cells after a pelvic examination, and cervical samples were collected into a prepared liquid medium (ThinPrep, Hologic, Marlborough, MA, USA). All collected samples were placed in a 4°C refrigerator and transferred to a third-party laboratory, KingMed Diagnostics, Kunming branch, for Cobas 4800 HPV testing (Roche, Basel, Switzerland) and Sansure PCR HPV test (Sansure Biotech, China). Sansure PCR HPV test (Zhao et al., 2019) is a PCR-based pioneered One-step Fast Release technology and has been approved by European Union Certificate. The test uses real-time fluorescent quantitative PCR to target and report 15 high-risk HPV types, including HPV-16, 18, 31, 33, 35, 39, 45, 51, 52, 53, 56, 58, 59, 66, and 68. Cycle threshold (Ct) values ≤ 39 is considered HPV positive and >39 is considered negative. Then the cytology slide was prepared and the cytological interpretation was made by experienced cytologists according to the Bethesda 2014 classification system (Diane Solomon, 2015).

### Detection of EBV

Total DNA was extracted from exfoliated cervical cells from liquid medium (ThinPrep) using a QIAamp DNA Mini Kit (Qiagen, Germany) according to the protocol recommended by the manufacturer. To detect EBV, 5 µl of DNA was then used for subsequent RT-PCR (Premix Ex Taq, Takara, Janpa) with a CFX96 Real-Time PCR Detection System (Bio-Rad, USA). In addition, β-globin primers were used to verify the quality of the DNA and exclude false negative results, and a positive control was included in each RT-PCR assay to ensure the success of the reaction. RT-PCR was performed for 45 cycles of amplification, and samples were considered negative if the cycle threshold (Ct) value was greater than 40. The primers and probes used in this study are identical to those in previous studies (Lo et al., 1998; Wadowsky et al., 2003).

### Colposcopy Examination and Pathology

Women positive for HPV on Cobas or Sansure PCR testing or with a cytological diagnosis of atypical squamous cells of undetermined significance (ASCUS) or higher (ASCUS+) were referred for a colposcopy examination. A directed biopsy was collected if any abnormality was identified under colposcopy. Endocervical curettage was performed if the squamocolumnar junction was invisible. Biopsy tissues were immediately immersed in 10% buffered formalin and transported to KingMed Diagnostics, Kunming branch for processing (fixed colposcopy-directed biopsy samples were dehydrated in graded ethanol, embedded in paraffin and sectioned, and hematoxylin and eosin (HE) staining was conducted according to standard protocols) and diagnosis by experienced pathologists blinded to other screening results. Pathology results were reported as negative, cervical intraepithelial neoplasia grade 1 (CIN1), grade 2 (CIN2), grade 3 (CIN3), microinvasive carcinoma (MIC), squamous cell carcinoma (SCC), adenocarcinoma *in situ* (AIS), or adenocarcinoma (ADC). Women with negative results by all tests (cytology <ASCUS and without HPV detection) were considered to be negative for the outcome of CIN2 or worse (CIN2+). Women with confirmed CIN2+ were recommended for treatment.

### Transcriptional Profiling and Analysis

#### Sample Preparation

Total RNA was extracted from formalin-fixed paraffin embedded (FFPE) biopsy tissues using the RNeasy FFPE Kit (Qiagen, Germany) according to the manufacturer’s instructions. RNA purity was evaluated using a NanoPhotometer spectrophotometer (Implen, USA), and integrity was assessed using an RNA Nano 6000 Assay Kit with a Bioanalyzer 2100 system (Agilent Technologies, USA). Next, the RNA concentration was measured using a Qubit RNA Assay Kit with a Qubit 2.0 Fluorometer (Life Technologies, USA). A total amount of 1 µg of RNA per sample was used to generate sequencing libraries. Briefly, mRNA was purified from total RNA using poly-T oligo-attached magnetic beads. Fragmentation was carried out using divalent cations at an elevated temperature in First Strand Synthesis Reaction Buffer. Then, a random hexamer primer and mRNA fragments as templates were used to synthesize the first-strand cDNA. Second-strand cDNA fragments were obtained using DNA Polymerase I and dNTP. Remaining overhangs were converted into blunt ends *via* exonuclease/polymerase activities. The 3’end adenylated and adaptor-ligated cDNA were then purified with AMPure XP system (Beckman Coulter, USA) to select the fragments with the correct target size (370-420 bp). Finally, the cDNA libraries were enriched by PCR amplification with Phusion High-Fidelity DNA polymerase, universal PCR primers and Index (X) Primer. The library quality was assessed on an Agilent Bioanalyzer 2100 system.

Before sequencing, clustering of the index-coded samples was performed on a cBot Cluster Generation System using a TruSeq PE Cluster Kit v3-cBot-HS (Illumina) according to the manufacturer’s instructions. Then, the library preparations were sequenced on an Illumina HiSeq platform, and 150 bp paired-end reads were generated.

#### Quality Control and Read Mapping

Clean reads were obtained by removing reads containing adapters, reads containing poly-N and low-quality reads from the raw data. All subsequent analyses were based on filtered reads. Paired-end filtered reads were mapped to the homo sapiens sequence (ftp://ftp.ensembl.org/pub/release-98/fasta/homo_sapiens/) with HISAT2 v2.0.5.

#### mRNA Expression Analysis

FeatureCounts v1.5.0-p3 was used to count the read numbers mapped to each gene. To normalize the read count, fragments per kilobase of transcript per million mapped fragments (FPKM) were calculated for each gene. Then, the DESeq R package (1.16.1) was utilized to perform differential expression analysis of the two groups. The resulting P values were adjusted using Benjamini and Hochberg’s approach for controlling the false discovery rate. Genes with an adjusted P value <0.05 or | log2-fold change | >0 were considered differentially expressed. Gene Ontology (GO) enrichment analysis of differentially expressed genes (DEGs) was implemented by the GOseq R package. GO terms with corrected P values less than 0.05 were considered significantly enriched by DEGs. Subsequently, we used the clusterProfiler R package to test the statistical enrichment of DEGs in KEGG pathways. The mRNA-seq data for this study can be found in the Sequence Read Archive (SRA) (the BioProject accession number is PRJNA701124, and the SRA records will be accessible with the following link upon publication: https://www.ncbi.nlm.nih.gov/sra/PRJNA701124).

### Quantitative Real Time-PCR (qRT-PCR)

To validate the results from mRNA-seq, 14 DEGs were selected for qRT-PCR test. The involved genes and primers are shown in **Table 1**. RNA samples were the same as the ones used for mRNA-seq. qRT-PCR was performed using One Step SYBR PrimeScript PLUS RT-PCR kit (Takara, Japan) on a CFX96 Real-Time PCR Detection System (Bio-Rad, USA). The reaction was performed as follows: 42°C for 5 min and 95°C for 10 s, followed by 40 cycles of 95°C for 5 s and 60°C for 30 s. The dissociation curve analysis were used to confirm the specificity of amplification of each product and the absence of primer dimer. Each sample was performed in triplicates. The *GAPDH* gene was used as a reference gene. The relative expression level of each gene was measured by the method of 2^-ΔΔCt^ and was standardized by reference gene.

**Table 1:** Sequences of RT-PCR primers used for validation.

Gene	Forward primer	Reverse primer
**C1QA**	GTCATCACCAACCAGGAAGAACCG	GGAGACGATGGACAGGCAGATTTC
**C1QB**	AGGTGAATCGGGAGACTACAA	CACTGCGGGGCTCATAATTG
**C7**	CTGAGTGGAAATGTCCTGTCC	CGCTTCCGACTAGATGATGTGT
**TFEC**	GAGCCCGAGAATTGGAACACAGAC	ATCAACCGTGCCAAGTGAAGCC
**SMC1B**	TCACTGCCATTGTTGTAGCCTCTG	GCGAGGAATGTCTCAGGTTCAGC
**CACNG4**	TCTGGTCTGTGGCGGGTGTG	GCTGTCGTGGTCGTAGTCATTGTC
**CDKN2A**	GATCCAGGTGGGTAGAAGGTC	CCCCTGCAAACTTCGTCCT
**SCGB3A1**	GCACCCTCAACCCGCTGAAG	ACACACTTCTGGGAGCCCTCTATG
**SLC13A5**	TACATGAAGGACACCAACATGC	GCGATCCTCTTGTGCAGGT
**RPTN**	TGAGTCACAAATCTACCAGTGGC	ACTGTCCACAATAAGAGCCTGAT
**PTGS2**	ATGCTGACTATGGCTACAAAAGC	TCGGGCAATCATCAGGCAC
**SLPI**	GCCTGGATCCTGTTGACACC	AAACGCAGGATTTCCCACAC
**SLC5A1**	TGGCGGCGGTGATTTACAC	TCCCACTTCGTGAAAAGCAAA
**CD55**	AGAGTTCTGCAATCGTAGCTGC	CACAACAGTACCGACTGGAAAAT
**GAPDH**	ACAACTTTGGTATCGTGGAAGG	GCCATCACGCCACAGTTTC

### Data Analysis in Public Database

We utilized GEPIA2 to access and analyze the public database on cancer tissues generated by The Cancer Genome Atlas (TCGA) program and normal tissues from the Genotype-Tissue Expression (GTEx) project.

### Immunofluorescence (IF) Assays and Western Blotting (WB)

IF was performed on FFPE sections after heat-induced antigen retrieval. Briefly, sections were blocked for 1 h, incubated overnight at 4°C with a rabbit primary antibody against human CACNG4 (Immunoway, YM3404), and subsequently incubated with a secondary Alexa Fluor 568-conjugated goat anti-rabbit IgG antibody (Abcam, ab175696) for 1 h. Slides were mounted with mounting medium containing DAPI and imaged by a Leica SP8 laser scanning confocal microscope system. For WB, samples were pooled and extracted using the FFPE Total Protein Extraction Kit (Sangon Biotech, C500058) and then incubated with primary antibody against CACNG4 or β-actin (Cell Signaling, #3700). The proteins were visualized using HRP-conjugated secondary antibody (Cell Signaling, #7040 or #7076) and chemiluminescent HRP substrate (Bio-Rad).

### Statistical Analysis

Descriptive statistical analysis was performed to summarize the prevalence of HPV and EBV infections and coinfections. Chi-square tests were used to compare the distribution of CIN2+ between the HPV infection and HPV-EBV coinfection groups. Student t-test was performed to evaluate the mRNA expression difference between two groups. *p* values less than 0.05 (two-sided) were considered statistically significant. Data analyses were performed using SPSS 20.0.

## Results

### Prevalence of HPV and EBV Infections and Coinfections in HIV-Positive Women

A total of 180 women were enrolled in this study; of these, four women with extremely high HIV viral load (≥10^6^ copies/ml) that reflected severe immunosuppression were excluded to avoid interference. Of 176 women, the median age was 40 (34–46) years. Among them, 61.9% have been married, 68.8% had education at junior high school or below, 45.4% were unemployed and 44.3% were farmer or rural migrant worker. The median age at sexual debut and the first delivery were 20 (19–22) and 24 (21–27) years, respectively. The median CD4 count was 540 (IQR 398–656) cells/µL, and the median HIV viral load was <50 (IQR <50–<50) copies/ml. Valid HPV test results were obtained from 176 women. The prevalence of HPV and EBV in HIV-positive women was 33.5% and 15.9%, respectively. In addition, the coinfection rate of EBV-HPV was 9.1%. Eleven participants were missing from the histopathological analyses due to loss to follow-up. Thus, a total of 165 women were involved in the distribution analysis of CIN2+ among different coinfection statuses, and **Table 2** shows detailed information on the STI distribution among these women. **Table 3** shows that EBV-HPV coinfected women were more likely to be diagnosed with CIN2+ (*p*=0.017) than those without coinfection.

**Table 2:** Detailed information on HPV and EBV distribution among 165 HIV-positive women.

HPV	EBV	ASCUS+ (n)	<ASCUS (n)	Total (N)	CIN2+ (n)	<CIN2 (n)^a^	Total (N)
**+**	**-**	4	39	43	1	36	37
**-**	**+**	0	12	12	0	11	11
**+**	**+**	3	12	15	4	10	14
**-**	**-**	2	102	104	0	103	103
**Total (N)**		9	165	174	5	160	165

a Besides the number of women were referred to colposcopy, the number of cases presented in this column also include the number of women that were ASCUS and HPV negative and were not referred to colposcopy.

**Table 3:** Distribution of CIN2+ in HPV- and HIV-positive women with or without EBV.

		CIN2+			
**EBV infection status**	**N**	**n**	**%**	**χ^2^**	***p***
With EBV	14	4	28.6	7.686	0.017
Without EBV	37	1	2.7		

Chi-square tests, p<0.05 indicates statistical significance. N, the number of HPV-positive women; n, the number of women with CIN2+.

### Comparison of General mRNA Expression Profiles Between Different Infection Statuses in HIV-Positive Women

**Figure 2:**
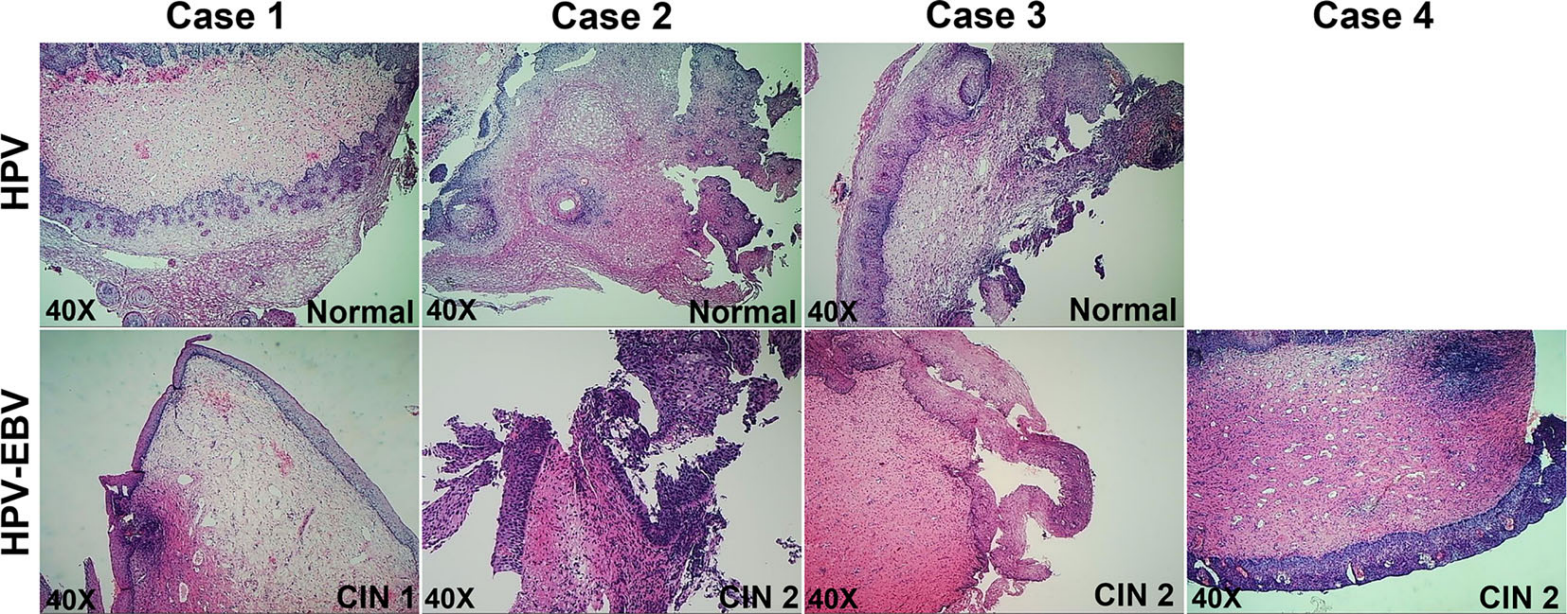
Pathology of cervical tissues involved in mRNA-seq.

**Figure 3:**
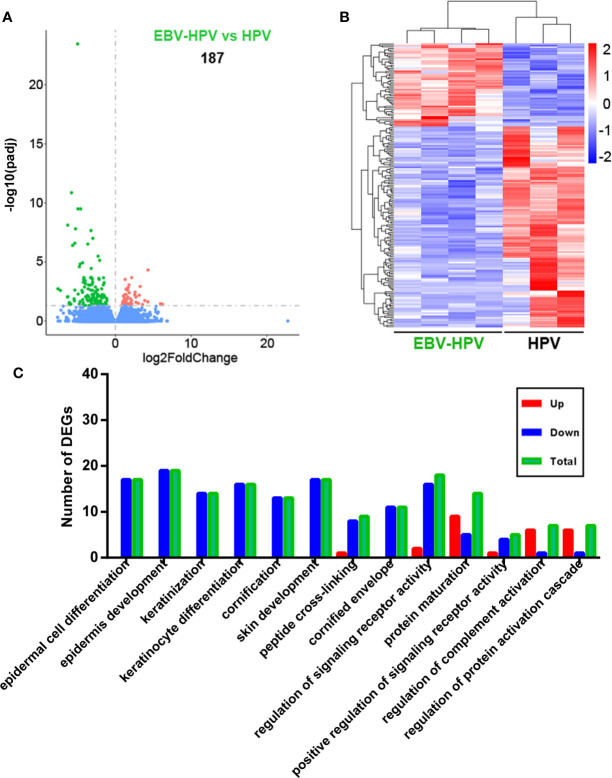
Analysis of cervical tissue transcriptome data of different viral infection statuses. **(A)** Differences in gene expression in the EBV and HPV coinfection groups compared with the HPV infection alone group (red and green dots indicate DEGs); the numbers in plots indicate the numbers of DEGs. **(B)** Hierarchical cluster analysis of DEGs within different paired comparisons confirming that patients with each infection or coinfection status could be grouped together based on similar gene expression profiles (red, upregulated; blue, downregulated). **(C)** Numbers of DEGs belonging to numerous significantly altered GO enrichment terms (up, upregulated DEGs; down, downregulated DEGs; total, total DEGs).

To clarify the reasons behind the significant correlation of EBV and HPV coinfection and CIN2+, we used mRNA-seq to capture the transcriptome changes in pathological tissues from colposcopy-directed biopsy samples. Owing to the relatively small patient number for biopsy, 7 samples within two groups were included in this analysis. The individual demographical and clinical characteristics are presented in **Supplementary Table 1**. The CIN grade and histopathology of these samples are presented in **Figure 2**. Total mRNA from the coinfection group was compared to the HPV alone infection group, revealing that there were 187 significantly altered genes (**Figure 3A**). The resulting heatmap confirmed that patients with the same infection status could be grouped together based on similar gene expression profiles (**Figure 3B**). Next, a GO analysis was used to identify potential biological or molecular mechanisms that were significantly altered in the coinfection group. The most significantly enriched common GO terms were associated with epidermal cell and keratinocyte differentiation, skin and epidermis development, and peptide cross-linking (**Figure 3C**). Most genes involved in these significantly changed GO terms were downregulated in the coinfection condition. However, several upregulated DEGs were significantly enriched in biological process GO terms concerning the regulation of signaling receptor activity, protein maturation, positive regulation of signaling receptor activity, regulation of complement activation and protein activation cascade. These findings suggest that coinfection of EBV and HPV may cause increased virus-host interactions and have a greater effect on host immunity.

### Specific DEGs and Overexpression of CACNG4 in EBV-HPV-Associated CIN Lesions

**Figure 4:**
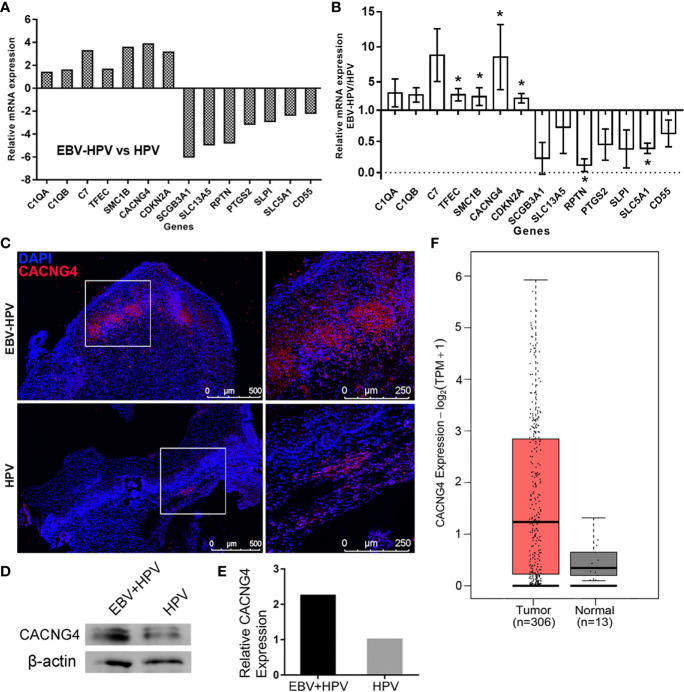
Selected mRNAs with significant changes and overexpression of CACNG4 in EBV-HPV-associated CIN lesions. Expression levels of the selected, significantly altered mRNAs of the mRNA-seq analysis **(A)** and RT-PCR validation **(B)**. Each value was normalized to the GAPDH value and is presented as the mean ± SD, *p<0.05, compared with the HPV group. **(C)** A red fluorescence signal indicating the presence of CACNG4 was detected in cervical tissues. **(D)** Protein expression of CACNG4 relative to β-actin expression in cervical tissues. **(E)** Densitometric analysis of the immunoblots. The intensity of each band was normalized to the β-actin intensity. Due to the limited amount of clinical material, the EBV-HPV sample consisted of 4 pooled biological replicates and the HPV sample consisted of 3 pooled biological replicates in **(D, E)**. **(F)** The expression of *CACNG4* between cervical cancer samples and normal tissue samples taken from TCGA and the GTEx databases. TPM, transcripts per million.

By combining the findings from the identified DEGs and the GO analysis, we selected 14 DEGs that were included in the enriched GO terms related to EBV and HPV coinfection or were the most up- or downregulated DEGs. RT-PCR was performed to validate the expression profiles of these marker genes in the EBV-HPV group, and the results were consistent with the mRNA-seq data (**Figures 4A, B**). Notably, a significant increase in *CACNG4* expression was observed among these genes. CACNG4, an L-type voltage-gated calcium channel gamma subunit, has been reported to be overexpressed in several cancers (Kuznetsova et al., 2007; Zhang et al., 2016; Slattery et al., 2018) and may promote calcium homeostasis to increase the survival and metastatic ability of tumor cells (Kanwar et al., 2020). This alteration in *CACNG4* expression may be linked to CIN lesions. IF (**Figure 4C**) and WB (**Figures 4D, E**) analyses were further performed and confirmed the specific upregulation of CACNG4 in EBV-HPV-coinfected CIN lesions at the protein level. Next, we examined the levels of *CACNG4* mRNA transcripts within cancer samples using the GEPIA2 website. A comparison of *CACNG4* expression between tumor samples and normal tissue samples taken from TCGA and the GTEx databases showed that *CACNG4* is upregulated in cervical cancer (**Figure 4F**). Taken together, these data suggest that CACNG4 may act as a potential biomarker for the early diagnosis of cervical cancer, as well as a target for modulating intracellular calcium homeostasis, which sustains tumor growth.

## Discussion

In this study, we showed the prevalence of HPV and EBV as single infections or coinfections among women living with HIV, and our results were similar to what has been previously reported (Camargo et al., 2014; Jin et al., 2017). Our findings also support that HIV-infected individuals have an increased risk for EBV infection compared with the general population (Petrara et al., 2012; Jin et al., 2017). Given that only a small percentage of HPV-positive women develop cancer, HPV is necessary but insufficient for carcinogenesis. Thus, mucosally transmitted viral cofactors appear to contribute to HPV-related cervical cancer. Human herpesvirus EBV shares similar routes and sites of infection with HPV. Genital coinfection of these viruses may disrupt the mucosal epithelial barrier (Hebner and Laimins, 2006) and the different viruses may influence each other in many ways, such as viral entry and clearance (Blanco et al., 2020). Consistent with previous reports (Santos et al., 2009; Kienka et al., 2019; Cameron et al., 2020; Joharinia et al., 2020), we revealed a significant association between EBV and HPV coinfection and CIN2+, suggesting that coinfection of EBV and HPV is a potential risk factor in carcinogenesis.

While there is a close relationship between EBV infection and HPV-related cervical cancers, the underlying mechanisms remain unclear. Several previous studies have examined gene expression changes in HPV-infected cell lines or tissues (Wong et al., 2006; Klymenko et al., 2017). However, no studies have analyzed the features of host gene expression in the presence of other viruses coinfected with HPV. In this study, exfoliated cells from the cervix were utilized to determine the viral infection status, followed by evaluating the global changes in the biopsy tissue using mRNA-seq. Due to the limited samples, we did not detect the viruses in these biopsy tissues. However, of particular interest is how abnormal host immune status induced by viral coinfections modulates epithelium gene expression. Based on our data, we revealed that obvious changes in the transcriptome in CIN tissues with EBV and HPV coinfection compared to normal tissues with HPV infection alone. Similar to previous transcriptomic studies (Klymenko et al., 2017) showing that HPV infection alone in epithelial cell lines abrogates differentiation and epithelial barrier formation, the current study also showed decreased epithelial and keratinocyte differentiation factors in the coinfection groups. For example, small proline-rich repeat (SPRR) protein family members, including *SPRR2A*, *SPRR2B*, *SPRR2D*, *SPRR2E*, *SPRR2F*, and *SPRR2G*, the late cornified envelope (LCE) gene cluster members *LCE3D* and *LCE3E*, and a number of keratin (KRT) protein-coding genes, such as *KRT17, KRT23* and *KRT24*, were downregulated (**Supplementary Table 2**). These findings suggest that coinfection of EBV and HPV increased the effect of HPV on epithelial differentiation and development, but these changes may mainly show a profile expected of viral infection rather than tumor progression. Meanwhile, the presence of virus and viral gene expression is unknown in the mRNA-profiled specimens and the histopathology grade of the HPV+ and HPV+EBV+ samples are not matched, so the findings are compelling but further studies are needed to confirm that EBV is responsible for the gene expression changes observed.

Intriguingly, several DEGs involved in EBV and HPV coinfection attracted our attention. The GO analysis indicated that some DEGs, including *CACNG4*, *C7*, *C1QA*, *C1QB*, *CD55*, and *SCGB3A1*, were involved in the immune response, signaling receptor activity, and protein activation, suggesting that coinfection with EBV and HPV may cause interactions with the host. In particular, some of these DEGs are closely associated with cancer. For example, genes related to the GO term “components of complement,” such as *C1QA*, *C1QB*, and *C7*, may promote tumorigenesis in cells within the tumor microenvironment (Bulla et al., 2016). *TFEC* is a member of the microphthalmia (MiT) family, which regulates multiple physiological processes, including cell survival, differentiation, proliferation, invasion, metabolism, and DNA damage repair, thus contributing to the initiation and development of some cancer types (Haq and Fisher, 2011; Goding and Arnheiter, 2019). We confirmed these changes in mRNA levels (**Figures 4A, B**); unfortunately, due to the limited amount of clinical material, we could not evaluate all of their protein expression levels. We therefore selected *CACNG4* as a candidate to evaluate its protein levels because of the highest upregulation of *CACNG4* mRNA levels (mean fold change=8.55). Indeed, a consistent upregulation of CACNG4 was found in cervical tissues (**Figures 4D, E**). Of interest, *CACNG4* encodes an L-type voltage-gated calcium channel (VGCC) γ4 subunit. One gene expression analysis revealed upregulation of the *CACNG4* gene in human colon cancer (Slattery et al., 2018). Breast tumors with nodal metastases are also associated with higher protein expression of CACNG4 in clinical samples (Kuznetsova et al., 2007). Mechanistically, CACNG4 affects calcium influx by modulating VGCCs, which in turn regulate the homeostasis and metastasis of tumor cells (Kanwar et al., 2020). Since VGCCs of different types have been associated with several cancers (Monteith et al., 2012), the possible oncogenesis mechanisms are seemingly linked to calcium-dependent mitogenic signals of epidermal growth factor (Huang et al., 2004). As such, CACNG4 may serve as a potential predictor for tumorigenesis in cervical disease. Nevertheless, considering that the EBV-HPV coinfected cases involved in mRNA-seq all had CIN lesions in this study, we were unable to determine whether gene expression changes were simply associated with coinfection status but not cervical lesions. However, we noted that EBV-HPV coinfected women were more likely to be diagnosed with CIN2+ (p = 0.017) than women infected with HPV alone, and the precise mechanisms underlying the association between herpesvirus and HPV coinfection and carcinogenesis await further investigation in the future.

In conclusion, we reported transcriptional changes in pathological tissues from HIV-positive women with EBV and HPV coinfection. Coinfection is associated with precancerous lesions, which leads to changes in the gene expression of epithelial differentiation and development compared to normal tissues with HPV only. More important, several cancer-related DEGs were involved in CIN lesions with coinfection. These findings provide some evidence that EBV can act as a cofactor or mediator in HPV-related cervical cancer. Notably, specific genes or proteins, such as CACNG4, may serve as biomarkers that can risk stratify patients based on pathological changes in the cervix. However, the relatively small sample size is the limitation of our study. Further large-scale studies of the general population will allow us to validate and select specific biomarkers for early intervention in cervical cancer.

## Data Availability Statement

The datasets presented in this study can be found in online repositories. The names of the repository/repositories and accession number(s) can be found below: https://www.ncbi.nlm.nih.gov/bioproject/PRJNA701124.

## Ethics Statement

The studies involving human participants were reviewed and approved by Ethical Review Committees of National Cancer Center/Cancer Hospital, Chinese Academy of Medical Sciences and Peking Union Medical College. The patients/participants provided their written informed consent to participate in this study.

## Author Contributions

MF, RD, and YG performed the experiments and analyzed the data. HZ analyzed the data and assisted with writing the manuscript. YQ assisted with the project design and revised the manuscript. QL and FZ designed the project. MF, RD, QL, and FZ wrote the manuscript. All authors contributed to the article and approved the submitted version.

## Funding

This work was supported by the Science and Technology Major Project of Yunnan Province (2017ZF006), the CAMS Initiative for Innovative Medicine (2016-I2M-1-019), and the National Natural Sciences Foundation of China (81761128006 and 31670173).

## Conflict of Interest

The authors declare that the research was conducted in the absence of any commercial or financial relationships that could be construed as a potential conflict of interest.

## Publisher’s Note

All claims expressed in this article are solely those of the authors and do not necessarily represent those of their affiliated organizations, or those of the publisher, the editors and the reviewers. Any product that may be evaluated in this article, or claim that may be made by its manufacturer, is not guaranteed or endorsed by the publisher.
