# Supplementary material for: Role of Epstein-Barr Virus and Human Papillomavirus Coinfection in Cervical Intraepithelial Neoplasia in Chinese Women Living With HIV
Source: Front Cell Infect Microbiol. 2021 Sep 7;11:703259. doi: 10.3389/fcimb.2021.703259 (PMC8453025; doi:10.3389/fcimb.2021.703259)
Supplement: Supplementary file 1 [file DataSheet_1.pdf]

Supplementary Table 1. The individual demographical and clinical characteristics

Group	Sample Name	Age, years old	Marriage status	Age of sexual debut	Age at first delivery	CD4 count, cells/μL	HIV viral load, copies/ml	HPV genotype	Histopathology grade
HPV	H1	38	married	20	N/A	265	0	52	normal
	H2	41	unmarried / divorced	24	24	360	24	52	normal
	H3	48	married	20	21	273	0	52	normal
EBV-HPV	H-E1	42	married	20	30	628	0	52,53,58	CIN 1
	H-E2	57	unmarried / divorced	19	25	Unknown	Unknown	45,52,58	CIN 2
	H-E3	27	unmarried / divorced	16	N/A	314	Unknown	33,39	CIN 2
	H-E4	34	married	17	21	558	0	16,33,39,68	CIN 2
