# Supplementary material for: Role of Epstein-Barr Virus and Human Papillomavirus Coinfection in Cervical Intraepithelial Neoplasia in Chinese Women Living With HIV
Source: Front Cell Infect Microbiol. 2021 Sep 7;11:703259. doi: 10.3389/fcimb.2021.703259 (PMC8453025; doi:10.3389/fcimb.2021.703259)
Supplement: Supplementary file 2 [file DataSheet_2.pdf]

Supplementary Table 2

Gene	Log2 Fold Change HPV-EBV vs. HPV	*P* value	Description
SPRR2A	-1.998	2.58E-05	small proline rich protein 2A
SPRR2B	-5.223	6.39E-30	small proline rich protein 2B
SPRR2D	-2.784	3.08E-06	small proline rich protein 2D
SPRR2E	-3.020	0.0055	small proline rich protein 2E
SPRR2F	-4.807	0.0001	small proline rich protein 2F
SPRR2G	-6.052	1.73E-05	small proline rich protein 2G
LCE3D	-4.996	5.42E-06	late cornified envelope 3D
LCE3E	-4.703	0.0030	late cornified envelope 3E
KRT17	-3.338	0.0032	keratin 17
KRT23	-2.810	5.01E-05	keratin 23
KRT24	-6.490	0.0003	keratin 24
